# Empathy for wildlife: The importance of the individual

**DOI:** 10.1007/s13280-024-02017-4

**Published:** 2024-05-25

**Authors:** Pauline Smith, Janet Mann, Abigail Marsh

**Affiliations:** 1 Environmental Justice Program, Earth Commons Institute, Georgetown University, Washington, DC 20057 USA; 2 Department of Biology and Department of Psychology, Earth Commons Institute, Georgetown University, Washington, DC 20057 USA; 3 Department of Psychology, Interdisciplinary Program in Neuroscience, Interdisciplinary Program in Cognitive Science, Georgetown University, Washington, DC 20057 USA

**Keywords:** Bottlenose dolphins, Charismatic and umbrella species, Empathy, Identifiable victim effect, Individual recognition, Pro-environmental behavior

## Abstract

Because climate change and the biodiversity crisis are driven by human actions, determining psychological mechanisms underpinning support for environmental action is an urgent priority. Here, we experimentally tested for mechanisms promoting conservation-related motivation and behavior toward a flagship species, wild Tamanend's bottlenose dolphins. Following evidence that empathy increases prosocial motivations and behavior, and that the ability to identify individual humans promotes empathy, we tested whether this relationship applied to the ability to identify individual dolphins. Participants identified dolphins from their dorsal fins at above chance levels, and better individuation correlated with higher empathy for dolphins and higher willingness to pledge environmental behaviors. Pairing a narrative with an image of an injured dolphin leads to higher donations relative to a narrative alone. Our novel finding that the ability to individually identify dolphins relates to empathy and conservation-related behavior suggests pathways for strengthening environmental attitudes and behavior.

**Supplementary Information:**

The online version contains supplementary material available at 10.1007/s13280-024-02017-4.

## Introduction

Anecdotally, it is well known among biologists that individual stories of charismatic megafauna have the power to dramatically shift public behavior–including the desire to help that species or ecosystem in general–in a way that generalized statistics and facts do not. For example, even while in the Northeast Pacific, the famous Southern Resident Killer Whale (SRKW) population has dwindled to the brink of extinction (Desforges et al. 2018), tens of millions of dollars were raised and invested in the failed attempt to return a single killer whale (Keiko, of “Free Willy” movie fame) to the wild (Orlean 2002). And in 2018, when a SRKW adult female, Tahlequah, carried her dead calf for weeks, she captivated the public and inspired the town of Tahlequah, Oklahoma, 2000 miles away, to establish the Oklahoma Killer Whale Project in partnership with a Washington state conservation organization (Curtis 2020). In the first instance, even if Keiko survived, no conservation values were served. In the second, the human response to the plight of a single whale galvanized real support for conservation measures. Both instances demonstrate the allure and emotional resonance of suffering of individual animals, particularly members of charismatic species, in motivating human action. But why? Answering this question hinges on understanding why humans respond to the suffering of wildlife in some, but far from all circumstances.

Broadly speaking, human activities are responsible for the current climate and biodiversity crisis. It follows then that mitigating these crises depends on a deep understanding of human behavior in relation to conservation of the natural world. Recently, conservation biologists and social–behavioral scientists have urged their communities to focus on the psychological underpinnings that drive people’s engagement with conservation and their support of it, through either donations, changes in personal behavior, or changes in attitude (Saunders 2003; Dalerum 2014; Selinske et al. 2018; Nielsen et al. 2021). Conservation psychology, defined as “the scientific study of the reciprocal role between humans and the rest of nature, with a particular focus on how to encourage conservation of the natural world,” is a growing discipline (Saunders 2003; Dietsch et al. 2020). However, it remains underrepresented in conservation journals (Selinske et al. 2018), and the basic psychological mechanisms that drive conservation-related behavior have received scant attention. Here we capitalize on and integrate established but discipline-specific phenomena that we hypothesize will be associated with human behavior favoring wildlife conservation: human interest in charismatic fauna (Di Minin and Moilanen 2014); the relationship between empathy and conservation-related behavior (Schultz 2000; De Berenguer 2007; Brown et al. 2019); and the relationship between individual recognition and empathy (Bate et al. 2010). Additionally, we experimentally investigate psychological phenomena associated with empathy and support for a charismatic flagship species, the Tamanend's bottlenose dolphin (*Tursiops erebennus*) (Albert et al. 2018; Costa et al. 2022), and whether the ability to recognize individual members of that species is associated with greater conservation intentions and behavior.

Identifying the underpinnings of psychological responses associated with conservation-related intentions and behaviors for “charismatic megafauna” may be of particular strategic value. These large, popular animals not only appeal to the public (Jepson and Barua 2015) and elicit more empathy (Young et al. 2018; Miralles et al. 2019) but attract donors to conservation organizations, several of which even use them as their mascots and logos (e.g., World Wildlife Fund uses pandas; Natural Resources Defense Council uses polar bears; and Oceana uses a stylized dolphin, see Colléony et al. 2017). Individuals within these charismatic species, such as Taliquah the killer whale, or Cecil the lion (Carpenter and Konisky 2019), can also be of particular interest for garnering interest and support for conservation and policy change (Jarić et al. 2023). Finally, charismatic species often occupy large ranges with many diverse species within, earning them the designation of “umbrella” species. Although conservation schemes designed to protect one species are rarely adequate for protecting all sympatric species (Andelman and Fagan 2000; Roberge and Angelstam 2004), charismatic umbrella species can support biodiversity and protect entire ecosystems if spatial overlap is sufficient (Caro and Riggio 2013; Higa et al. 2016; Yamaura et al. 2018).

While the conservation status of Tamanend’s bottlenose dolphins is not yet known (Costa et al. 2022), this genera occupies most coastal ecosystems, and protecting dolphins can be an effective surrogate for protecting other species in the same habitats (Sergio et al. 2008). Dolphins are not only highly charismatic (Albert et al. 2018), but are also familiar to the public, making them a good candidate for conservation fundraising (Colléony et al. 2017). Following the well-documented link between prosocial outcomes—including conservation-related prosocial outcomes such as donating to and reporting support for wildlife conservation (Ghasemi and Kyle 2021)—and empathy, we also sought to identify factors that promote empathy for bottlenose dolphins (hereafter, dolphins).

Empathy is defined as the internal representation or simulation of another’s sensory or affective state, which enables that state (e.g., fear or pain) to be identified and responded to appropriately (Marsh 2019). Empathy can be assessed using self-report or via empathic accuracy measures in which respondents are asked to identify if a target is experiencing pain or distress (Marsh 2022). Although distinct from care (also termed compassion or concern), empathy often leads to care, which in turn motivates prosocial behaviors (Lamm et al. 2019; Marsh 2019). Humans consistently respond empathically to the suffering and distress of other humans, domestic pets, and humanlike or humanized species (Colléony et al. 2017; Thompson 2024), but relatively little research explores empathic processes for wildlife (Taylor and Signal 2005). And several studies demonstrate that induced or dispositional empathy for nature influences conservation behavior, providing a promising avenue for further investigation (De Berenguer 2007, 2010; Tam 2013; Pfattheicher et al. 2015).

There are several potential barriers to empathizing with wildlife like dolphins. Empathy is typically elicited by obvious suffering or distress (Västfjäll et al. 2014; Marsh 2019). But suffering and distress may be difficult for human observers to interpret in species like dolphins, who are less visible because of their marine habitat and who do not share human nonverbal cues, such as facial expressions, to signal distress. In fact, for centuries the dolphin’s fixed “smile” has been misinterpreted as signaling happiness or friendliness (Devine and Clark 1967). Dolphin suffering or distress must thus be inferred from visible injuries or contextual cues like the presence of threats. Second, empathy is typically heightened for more familiar or similar others (Caviola et al. 2021), and dolphins’ outward appearance and behavior differ starkly from humans’.

Finally, individual dolphins may be difficult for untrained observers to identify. This is an important consideration in light of findings showing that the ability to individuate people (i.e., to tell them apart and recognize them as individuals) positively correlates with self-reported empathy and empathic accuracy (accurate recognition of states like pain or distress) for people (Bate et al. 2010; Minio-Paluello et al. 2020; Giannou et al. 2022). This may reflect the fact that individual recognition is enhanced for those individuals considered to be socially salient and personally significant (Bernstein et al. 2007), which are also features associated with increased empathy (Hein et al. 2010). This phenomenon is related to the well-established *identifiable victim effect*, according to which empathy is most robustly elicited by suffering in a specific, identifiable individual rather than by groups or abstract entities (Lee and Feeley 2016). Although trained scientists can reliably identify individual dolphins by their dorsal fins (Urian et al. 2015), whether non-expert observers can individuate wild dolphins is unknown. Moreover, whether the ability to recognize individual wild animals such as dolphins is associated with increased empathy for them has not yet been explored.

We thus aimed to test for the first time whether the ability to individuate members of a species is related to empathy and conservation-related motivation and behavior toward them. In three studies, we tested human non-expert observers’ ability to recognize individual dolphins from their dorsal fins and whether this ability predicted empathic responses to descriptions or images of an injured dolphin and various conservation-related motivations and behaviors. To explore potential causal mechanisms, we also assessed the effects of several manipulations aimed at increasing individuation and empathy, including pairing fins with human names. These studies are unique in that: (1) faces were not used to elicit empathy; (2) we test for the first time whether non-experts can individuate dolphins; and (3) we test whether this ability corresponds to empathy toward dolphins and real-world conservation outcomes.

## Materials and methods

### Pilot study

In order to assess the feasibility of this study and the ability of non-experts to recognize dolphin dorsal fins, a pilot study was conducted among 197 participants. Informed consent in this and subsequent studies was obtained from participants, and the study was approved by the Internal Review Board of the authors University (#2008-606).

### Stimuli

**Fig. 1:**
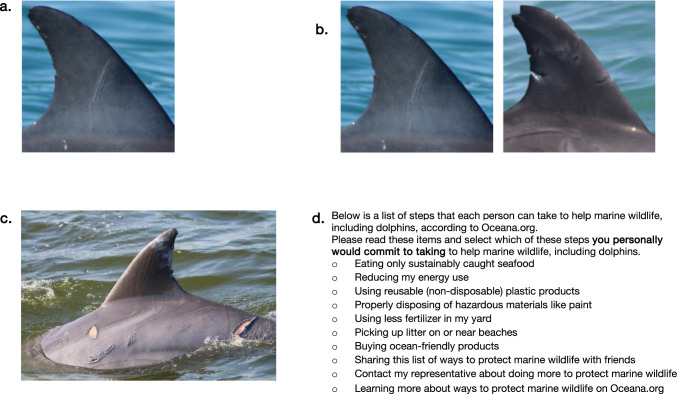
Elements shown to participants during the dolphin identification task and when presented with information about an injured dolphin, including (**a**) sample image shown at the beginning of the task, (**b**) sample image shown in the recognition phase of the task, (**c**) injured dolphin image and (**d**) real-world actions participants to which participants can commit. Images were taken under NMFS Permit #s: 19 403 and 23 782. Photo credit: Melissa Collier, Ann-Marie Jacoby, and the Potomac-Chesapeake Dolphin Project

Bottlenose dolphin dorsal fin images from the Potomac-Chesapeake Dolphin Project (pcdolphinproject.org) were selected that varied in image characteristics such as distinctiveness in shape and nicks–notches. All images were taken under NMFS permit no. 23782 and showed adult dolphins that have been individually identified by the Potomac-Chesapeake Dolphin Project team of scientists. All photographs were of a single fin with no other objects in view and were of high quality (in focus) with the dorsal fin (lateral view) taking up most of the frame (consistent with Urian et al 2015), with exception of one injured dolphin photograph where the injured lateral side was visible (Fig. 1c).

### Participants

Participants were recruited from Amazon’s Mechanical Turk (mTurk) between June 1, 2021, and January 14, 2022, and tested in Qualtrics (see Table 1 for demographic details). The pilot study, study 1, and study 2 recruited separate samples of participants.

**Table 1:** Socio-demographic data for each study and for all participants

	Study 1 (*N* = 399)	Study 2 (*N* = 667)	Total (*N* = 1066)
Gender (% women)	39.1%	46.5%	43.7%
Age (*M*, SD, range)	36.8 (10.1) [19–65]	37.6 (10.3) [18–65]	37.3 (10.3) [18–65]
Ethnicity (% white)	69.7%	84.9%	79.2%
Education level (% 4-year college degree)	74.2%	78.6%	77.0%
Employment status (% employed full-time)	80.2%	82.8%	81.8%

### Procedure

The recognition task we created was modeled on the recognition memory task for faces (RMF) used in neuropsychological testing in which respondents first view a series of faces and then, following a rest period, view each face again paired with a novel distractor face to assess the ability to identify previously seen individuals (Warrington 1984). In our task, participants first viewed 20 unique images of bottlenose dolphin fins successively, each for 6 s, in a randomized order (Fig. 1a). Participants were informed that they would be asked to identify these images later on. They then completed an intermediary task, the 28-item Interpersonal Reactivity Index (IRI) (Davis 1980), which is the most widely used self-report measure of empathy, and which contains an empathic concern subscale (Hall and Schwartz 2019; Kamas and Preston 2021). Participants next viewed a series of 20 pairs of photographs of dolphin fins. Each pair included one previously viewed fin and a second novel fin that was similar, shown facing the same direction. The novel fin photograph had an equal probability of being shown either on the left or on the right side of the screen. Participants were asked to indicate which of the fins they had previously viewed (Fig. 1b).

### Statistical analysis

Analyses were performed using R version 4.0.5. As normality assumptions were not met, we used nonparametric tests. Associations between variables were assessed using Spearman rank-order correlation coefficients and linear models that included gender, age, education, and knowledge about dolphins as covariates in addition to variables of interest (see Supplementary Figure 1). Multiple comparisons were controlled for using the Holm–Bonferroni method. Accuracy scores were compared to chance using exact binomial tests, and conditions were compared using Kruskal–Wallis *H* tests and Mann–Whitney *U* tests. (For all tests, very similar results were obtained using parametric tests.)

## Study 1: Individuation and empathy for dolphins

We first sought to test whether successfully individuating dolphins is associated with participants’ empathy for dolphins, willingness to commit to real-world actions to help marine wildlife, and donations to a marine conservation nonprofit (Oceana) selected for its 4-star rating on CharityNavigator.org.

In addition, we included a naming manipulation based on previous work demonstrating that pairing images with human names improves individuation and recognition of human faces (McGugin et al. 2011) as well as empathy for non-human entities (Vaes et al. 2016). In the memorization phase of the recognition task, participants were randomly assigned to view fins paired with human names (e.g., Edward, *N* = 131), non-human names (e.g., Asteroid, *N* = 129), or no names (*N* = 129).

### Procedure

After completing the identification task described above, participants (*N* = 399) viewed a photograph of a recently injured dolphin described as having been injured by a boat strike (Fig. 1c). This condition was based on the “outrage effect,” wherein anthropogenic harms to wildlife increase donations (Shreedhar and Mourato 2019) as well as greater empathy and a larger identifiable victim effect when victims are seen as blameless for their hardships (Shanahan et al. 2012; Lee and Feeley 2016). Participants rated how much fear they believed the dolphin felt when it was injured and how much pain and how much compassion they felt for the dolphin (0–10 scales).

Participants then were asked how much (up to $1.50) of their $2 payment they wished to donate to Oceana.org, described as a conservation nonprofit that benefits marine wildlife (all donated proceeds were sent to Oceana). They indicated their donation on a sliding scale from $0 to $1.50.

Participants next completed several measures aimed at assessing potential covariates, including a brief 10-item quiz assessing basic knowledge of dolphins (Supplementary Table 1), and provided demographic information, including gender, age, education, income, occupation, and race/ethnicity.

Finally, participants viewed 10 potential steps one could take to help marine wildlife, including dolphins, according to Oceana.org (e.g., eating only sustainably caught seafood or contacting their representative about protecting marine wildlife) (Fig. 1d). They selected which steps they personally would commit to taking.

### Results

As in the pilot study, participants recognized individual dolphins above chance levels (*M* = 0.65, SD = 0.16, range: 0.2–1.0, *p* < 0.001).

Across conditions, participants’ estimates of fear and pain and self-reported compassion for the injured dolphin were highly correlated (Cronbach *α* = 0.88) and thus were averaged to yield a composite empathy score (*M* = 7.4, SD = 2.1, range: 0–10). Empathy for the injured dolphin was positively correlated with trait-level empathic concern as measured by the IRI (rho = 0.47, *p* < 0.001).

Dolphin recognition was also positively correlated with empathy for the injured dolphin (rho = 0.31, *p* < 0.001). A planned multiple linear regression model found that the ability to recognize individual dolphins predicted greater empathy for the injured dolphin even controlling for gender, age, education, and knowledge about dolphins (*b* = 1.87, SE = 0.63, *t*(393) = 2.96, *p* = 0.003) (See Tables 2 and 3, for correlations between variables).

Across conditions, participants were willing to commit to an average of 3.5 real-world actions (SD = 2.7, range: 1–10). Dolphin recognition scores were also positively correlated with the number of real-world actions committed to (rho = 0.42, *p* < 0.001). A second planned multiple linear regression model found that recognition scores predicted the number of real-world actions that participants committed to, even when controlling for gender, age, education, and knowledge about dolphins (*b* = 4.90, SE = 0.78, *t*(393) = 6.26, *p* < 0.001).

**Fig. 2:**
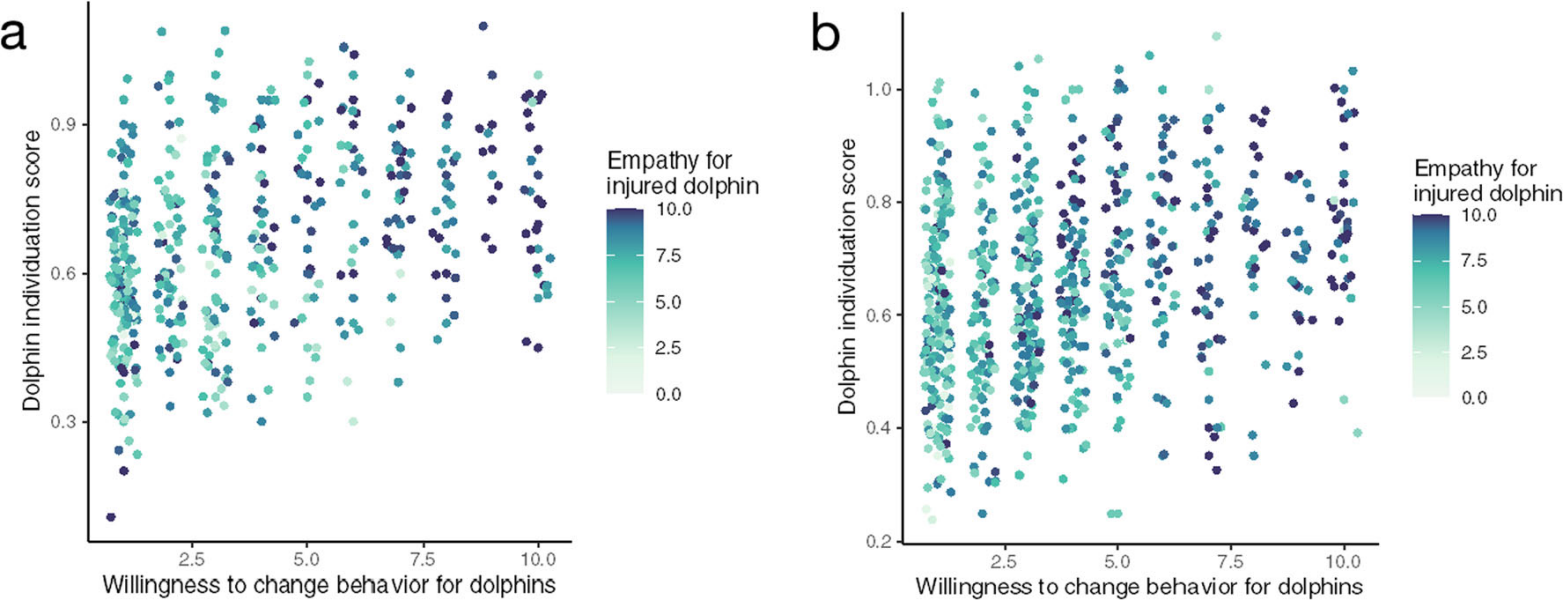
Empathy for the injured dolphin partially mediates the effect of dolphin recognition on the number of real-world actions participants pledged in study 1 (**a**) and study 2 (**b**). Dolphin individuation score (on a 10-point scale) is shown as a function of the number of actions participants pledged (out of 10 propositions), and color represents empathy for the injured dolphin (on a 10-point scale)

A bootstrap mediation analysis shows that empathy for the injured dolphin partially mediated the effect of dolphin recognition on the number of real-world actions participants pledged: Unstandardized indirect effects were computed for each of 1000 bootstrapped samples, and the 95% confidence interval was computed by determining the indirect effects at the 2.5th and 97.5th percentiles (*b* = 1.22, 95% CI [0.80, 1.76], *p* < 0.001) (Fig. 2).

Across conditions, 80.0% of participants donated to Oceana, with an average donation of $0.63 (SD = 0.51, range: 0.0–1.5). However, the dolphin recognition score was negatively correlated with donation amount (rho =  − 0.20, *p* < 0.001).

Kruskal–Wallis *H* tests comparing our three conditions found that viewing dolphin fins paired with names in the memorization phase did not impact recognition (*H*(2, *N* = 399) = 0.28, *p* = 0.9), empathy (*H*(2, *N* = 399) = 3.62, *p* = 0.2), real-world actions committed to (*H*(2, *N* = 399) = 0.13, *p* = 0.9), or donations (*H*(2, *N* = 399) = 0.20, *p* = 0.9).

## Study 2: Impact of individual narratives and photographs

### Procedure

The design and procedures for this study were identical to study 1, with the following exceptions. Following the null effect of naming, participants (*N* = 667, Table 1) did not view dolphins presented with names during the recognition task. Instead, during the empathy task, participants were randomized to one of five manipulations previously found to shape individuation and empathy. These five conditions varied across three dimensions: Information about dolphin injuries was either presented as information about dolphins in general or about a specific dolphin; this information was presented either as factual bullet points or through a short narrative account; and the information was either shown alone or paired with the photograph that was presented in study 1. We assessed these manipulations because both information about a specific individual and pairing information with a photograph of an individual are manipulations which can trigger the identifiable victim effect toward humans, so we expected them to heighten empathy and altruistic behavior (Lee and Feeley 2016; Thomas-Walters et al. 2020; Greving and Kimmerle 2021). The use of a narrative can also heighten empathy and encourage altruistic behavior, including in the context of environmental action (Kelly et al. 2014; Bruneau et al. 2015).

Participants were shown general information about dolphin injuries (*N* = 146) versus information about an individual dolphin’s injuries (*N* = 136), a narrative account of an individual injured dolphin (*N* = 140), the narrative account accompanied by the photograph from study 1 (*N* = 140), or only the photograph (*N* = 140) (Supplementary Table 2).

### Results

Across conditions, participants again individuated dolphins at above chance levels (*M* = 0.64, SD = 0.15, range: 0.25–1.0, *p* < 0.001). Participants’ estimates of fear and pain and self-reported compassion for the injured dolphin were again highly correlated (Cronbach *α* = 0.88) and thus were again averaged to yield a composite empathy score (*M* = 7.6, SD = 1.9, range: 1–10). Empathy for the injured dolphin was again positively correlated with trait-level empathic concern as measured by the IRI (rho = 0.49, *p* < 0.001).

The ability to individuate dolphins was again positively correlated with empathy for the injured dolphin (*M* = 7.6, SD = 1.9, range: 1–10; rho = 0.25, *p* < 0.001). This association remained positive when controlling for gender, age, education, and knowledge about dolphins in a planned multiple linear regression (*b* = 1.25, SE = 0.50, *t*(661) = 2.49, *p* = 0.01).

Across conditions, participants committed to an average of 3.5 real-world actions (SD = 2.5, range: 1–10). The ability to individuate dolphins was also again positively correlated with the number of real-world actions participants that were ready to commit to (rho = 0.29, *p* < 0.001), and this association was again positive when controlling for gender, age, education, and knowledge about dolphins (*b* = 2.55, SE = 0.64, *t*(661) = 3.95, *p* < 0.001). And again, empathy for the injured dolphin partially mediated the effect of dolphin individuation score on the number of real-world actions participants pledged, (*b* = 1.05, 95% CI [0.74, 1.44], *p* < 0.001).

Across conditions, 75% of participants donated money, and participants donated an average amount of $0.66 (SD = 0.57, range: 0.0–1.5). Again, the ability to recognize individual dolphins was negatively correlated with donations (rho =  − 0.21, *p* < 0.001).

**Fig. 3:**
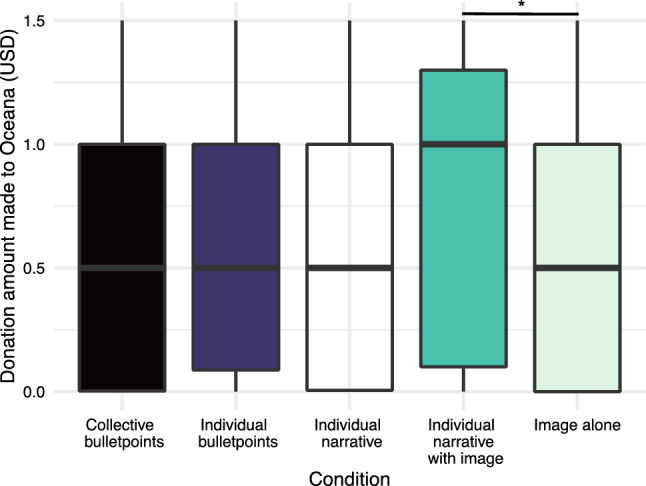
Distribution of donation amounts (in US dollars) from participants in each condition. Collective bulletpoints included general dolphin information and causes of injury. Individual bulletpoints listed details about the injured dolphin. The individual narrative described the injured dolphin in narrative form

Mann–Whitney *U* tests were conducted to assess the effect of the manipulations on outcomes of interest. Pairing a narrative about an individual dolphin with a photograph yielded higher donations to Oceana than the photograph alone (*W* = 10 835, *p* = 0.006; Fig. 3). Presenting individual versus general information did not affect donation behavior (*W* = 8597.5, *p* = 0.7), nor did presenting a narrative versus facts (*W* = 8547.5, *p* = 0.8), or presenting narrative with versus without a photograph (*W* = 8124, *p* = 0.2). We found no effect of any single manipulation on self-reported willingness to engage in real-world environmental actions or empathy.

## Discussion

Although bottlenose dolphins are not critically endangered, marine mammals are at the front lines of climate change, experiencing dramatic declines, and several species are on the brink of extinction (Albouy et al. 2020). Mechanisms that promote empathy and altruistic behavior toward dolphins might well be generalizable to other species and ecosystems.

Human recognition of individual members of wildlife species has clear scientific value. Over 67 long-term studies (10 + years) of wild cetaceans rely on individual recognition (photograph identification) to monitor populations (Mann and Karniski 2017). And dozens of long-term studies of wild primates, hyenas, elephants, zebra, red deer, pronghorn, bighorn sheep, albatross, etc. all follow individuals through time (e.g., Clutton-Brock and Sheldon 2010). But this is the first study to show that human recognition of individual members of a wildlife species may also have conservation value. We evaluated individual recognition of dolphins by non-experts, and we found that non-experts can recognize individual dolphins at a rate higher than chance. More importantly, we found that the ability to recognize individual dolphins is associated with greater empathy for them and greater willingness to commit to real-world environmental actions to protect them. While there has been some interest in photograph matching of animals, notably in the field of citizen science (Schofield et al. 2008; Austen et al. 2018; Aeluro and Oast 2022), very little research focuses on individual recognition of animals by humans (e.g., Phelps and Roberts 1994) and none on how this relates to empathy for wildlife.

These findings link to the growing literature on factors that promote empathy and prosocial behavior for humans. Parallel to our findings, the ability to recognize individual human faces correlates positively with empathy for humans (Bate et al. 2010) and feelings of common humanity and connectedness with them (Giannou et al. 2020). Relatedly, people tend to be worse at individuating the faces of members of out-groups and those to whom they feel less socially close (Ackerman et al. 2006; Bernstein et al. 2007). Our findings suggest that this phenomenon may apply across species as well—and to individual recognition based on features (fins) humans do not even possess. To our knowledge, this study is the first to link the ability to identify individual wild animals to empathy and conservation behavior intentions. Our recognition task was based on a classic face recognition memory task used in neuropsychological testing (Warrington 1984). More recently, newer face recognition tasks have been developed that assess social recognition across separate images of the same individual to ensure it is memory for the individual being tested, not specific visual features of an image. Because this kind of social recognition task can be extremely challenging even for human faces (Duchaine and Nakayama 2006; Jenkins et al. 2011), we elected not to use a similar paradigm and instead attempted to control for features of the presented images (e.g., all fins were depicted in isolation against water). However, a paradigm of this kind might enable more precise testing of correlates of the recognition of individual dolphins, including how human observers recognize individual dolphins and whether this ability can be improved (similar to previous studies of human recognition of individual monkeys, Dufour and Petit 2010).

Potentially relevant to this study is the small body of work exploring the identifiable victim effect for wildlife (Markowitz et al. 2013; Thomas-Walters and Raihani 2017). In one prior study, Thomas-Walters and Raihani (2017) found higher donations for flagship species than for non-flagship, but found no evidence of an identifiable victim effect. In another study, non-environmentalist students were more willing to donate to polar bears or pandas when told their donation would help a single identified animal compared to a group, although this effect did not occur in environmentalist students (Markowitz et al. 2013). Like our study, this research suggests individual differences are important to consider for understanding conservation-related behavior for charismatic species.

We also found that highly individuating information (a photograph of an injured dolphin paired with a narrative about it) yielded the largest monetary donations to a conservation organization. This specific difference had never been tested to our knowledge, but the result is consistent with previous findings showing that longer narratives can lead to more charitable donations in a medical context (Wu et al. 2022). Some previous studies had explored the impact of animal images on pro-environmental behavior (Thomas-Walters et al. 2020), showing that images of charismatic species and of injured animals can be particularly effective at eliciting donations. This highlights the importance of how information about a victim is presented (Lee and Feeley 2016; Shreedhar 2021), and underscores the impact of effective science communication more generally (Martell and Rodewald 2020; Shreedhar and Thomas-Walters 2022).

Other interventions did not affect the ability to individuate dolphins or empathy for them. Although pairing human faces with names can enhance face recognition and empathy (McGugin et al. 2011), we found no such effect for dolphins. In one prior study, human names enhanced empathy even for vegetables like carrots (Vaes et al. 2016). While carrots might *need* names to elicit empathy, images of injuries to a dolphin might induce such strong empathy that names have little additional impact. Alternatively, as suggested by Thomas-Walters and Raihani (2017), people may perceive the names of wild animals as contrived or arbitrary, unlike human or pet names.

**Table 2:** Empathy for the injured dolphin and real-world action pledged in all conditions of both studies,* M* (SD)

Condition	Study 1 (*N* = 399)				Study 2 (*N* = 667)					
	Human name	Object name	No name	Total	Facts (collective)	Facts (individual)	Narrative (individual)	Narrative (individual) + photograph	Photograph	Total
*N*	131	129	139	399	138	128	131	137	133	667
*Empathy, M (SD)*										
Fear	7.2 (2.2)	7.7 (2.4)	7.0 (2.3)	7.3 (2.3)	7.4 (2.2)	7.0 (2.0)	7.3 (2.2)	7.6 (2.0)	7.5 (2.1)	7.3 (2.1)
Pain	7.5 (2.1)	7.6 (2.4)	7.4 (2.4)	7.5 (2.3)	7.5 (2.3)	7.4 (2.0)	7.6 (2.2)	7.8 (1.9)	7.8 (2.1)	7.6 (2.1)
Compassion	7.7 (2.1)	7.5 (2.6)	7.5 (2.4)	7.6 (2.4)	7.5 (2.3)	7.5 (2.1)	7.5 (2.4)	7.8 (2.1)	8.2 (1.8)	7.7 (2.2)
Composite	7.5 (1.9)	7.6 (2.2)	7.3 (2.1)	7.4 (2.1)	7.5 (2.1)	7.3 (1.8)	7.4 (2.0)	7.8 (1.8)	7.8 (1.7)	7.6 (1.9)
Real-world actions (/10)	3.4 (2.7)	3.5 (2.7)	3.5 (2.7)	3.5 (2.7)	3.6 (2.6)	3.1 (2.2)	3.8 (2.7)	3.5 (2.6)	3.6 (2.5)	3.5 (2.5)
*Donations, M (SD)*										
% donating	78%	86%	76%	80%	75%	78%	75%	80%	68%	75%
Average donation (/$1.50)	$.84 (.43)	$.71 (.45)	$.85 (.46)	$.79 (.45)	$.88 (.50)	$.86 (.48)	$.89 (.49)	$.94 (.49)	$.83 (.47)	$.88 (.48)
Including null donations	$.65 (.52)	$.61 (.49)	$.65 (.54)	$.63 (.51)	$.88 (.50)	$.86 (.48)	$.89 (.49)	$.94 (.49)	$.83 (.47)	$.66 (.57)

Although participants donated slightly more than 40% of the maximum amount, consistent with the literature (Umer et al. 2022), we found that donations to Oceana were uncorrelated or even negatively correlated with other measures assessing empathic and prosocial outcomes as well as with identity recognition. This unexpected result was consistent across our two studies, suggesting the need for a deeper understanding of the underlying factors at play. Altruistic appeals and empathy inductions may generally be less successful at increasing monetary donations than other forms of altruism, like volunteering (Kim 2014; Marsh et al. 2007).

Alternately, participants might perceive real-world action as more effective than a small donation to a marine conservation organization, especially since Oceana may have been unfamiliar to our participants, and we did not specify how Oceana would help dolphins. This topic is worth further exploration given the importance of donations to the success of conservation initiatives.

**Table 3:** Summary of results for all variables of interest (mean and standard deviation). Scores for dolphin identification and empathic concern are normalized to be expressed out of 10, M (SD)

	Study 1 (*N* = 399)	Study 2 (*N* = 667)	Total (*N* = 1066)
Dolphin identification score (/10)	6.5 (1.6)	6.4 (1.5)	6.5 (1.5)
Knowledge about dolphins (/10)	6.9 (1.7)	6.9 (1.6)	6.9 (1.7)
Empathic concern (/28)	6.3 (1.6)	6.3 (1.8)	6.3 (1.7)
Empathy for injured dolphin (/10)	7.4 (2.1)	7.6 (1.9)	7.5 (2.0)
Donation (% of participants who donated money)	80.0%	75.3%	77.0%
Donation amount of those who gave money (/$1.50)	$ .79 (0.45)	$ .88 (0.49)	$ .85 (0.48)
Real-world actions pledged (/10)	3.5 (2.7)	3.5 (2.5)	3.5 (2.6)

Our fundamental goal was to identify mechanisms that promote empathy and conservation-related behavior and intentions toward dolphins, which might generalize to other species and ecosystems. Strategies promoting prosocial behavior toward dolphins can be applied to other marine mammals, and fundraising for dolphins can benefit other marine species, as they are a charismatic umbrella species for coastal ecosystems (Albert et al. 2018; Wells et al. 2004).

## Conclusions

The individual remains a key ingredient for eliciting empathy, even when knowledge about dolphins is controlled for. It may be that, at baseline, many people think about wild animals as undifferentiated collectives rather than as *individuals* with personalities, desires, and feelings that can motivate empathy and care. This may in part reflect poor knowledge of or exposure to wild animals, or feelings of threat from or competition with them (Kellert et al. 1987), any of which can suppress empathy and care. Individual identification, imagery, narrative and species charisma likely operate synergistically to arouse empathic concern for wildlife. Pairing these features with more convincing narratives might be effective in inducing the public to see wildlife as individuals and promote fundraising for conservation or encouraging sustainable behavior. While scientists often shy away from anecdotes, developing narratives to illustrate the impacts of human activity on wild animals is likely to be impactful. Policy must be data-driven, but moving hearts and minds to act rarely relies on statistics alone (Jacobson et al. 2007; Zebregs et al. 2015). News media has, of course, perfected this technique, often leading with an individual narrative and image to garner interest. As the first study of how such mechanisms elicit empathy for wildlife, we hope additional research can lead to strategies for strengthening public support for policies addressing the spiraling decline in biodiversity.

### Supplementary Information

Below is the link to the electronic supplementary material.

Supplementary file1 (PDF 420 KB)

## Data Availability

Data is available on OSF at the following URL: https://osf.io/jr3cb/.
