# Supplementary material for: Empathy for wildlife: The importance of the individual
Source: Ambio. 2024 May 25;53(9):1269–80. doi: 10.1007/s13280-024-02017-4 (PMC11300747; doi:10.1007/s13280-024-02017-4)

AMBIO

Electronic Supplementary Material
This supplementary material has not been peer reviewed.

Title: **Empathy for wildlife: The importance of the individual**
Authors: Pauline Smith, Janet Mann, Abigail Marsh.

Supplementary materials

Supplementary Table 1. Study 1 and 2 questions and answers testing general knowledge about dolphins

Question	Correct answer
There is only one species of dolphin	False
Dolphins are more closely related to whales than to sharks	True
Dolphins eat mostly plants	False
There are no freshwater dolphins	False
Dolphins are mammals	True
Male dolphins look after calves	False
Dolphin sonar is superior to the Navy sonar	True
Dolphins are social (spend most of their time with other dolphins)	True
Dolphin tails move from side to side when they swim	False
Dolphins communicate using squeaks, whistles and clicks	True

Supplementary Table 2. Study 2 condition details.

Condition	Details	Collective / individual	Facts / narrative	Image / none
Facts about general dolphin injuries	• Every year, thousands of dolphins are struck by boat traffic<br>• It is common to see dolphins with wounds or scars from boat-strike injuries<br>• Dolphins are susceptible to being struck by boats because they spend much of their time in coastal areas where boat traffic is high.	Collective	Facts	None
Facts about an individual dolphin's injuries	• ID: Naia<br>• Age: 14<br>• Species: Bottlenose<br>• Location: Chesapeake Bay<br>• Family structure: Mother and three siblings in same pod<br>• Markings: Two wounds on flanks caused by boat strikes in 2020	Individual	Facts	None
Narrative about an individual dolphin's injuries	Naia is a 14-year-old bottlenose dolphin who lives together with her mother, sister, and two brothers. She has pink gashes on her sides that are wounds caused by a boat striking her in 2020. Wounds like these are a common occurrence for dolphins like Naia who live in the Chesapeake Bay and other coastal areas where boat traffic is high.	Individual	Narrative	None
Narrative about an individual dolphin's injuries + photo	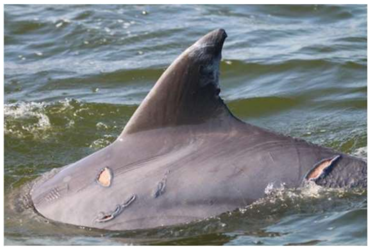<br>Naia is a 14-year-old bottlenose dolphin who lives together with her mother, sister, and two brothers. She has pink gashes on her sides that are wounds caused by a boat striking her in 2020. Wounds like these are a common occurrence for dolphins like Naia who live in the Chesapeake Bay and other coastal areas where boat traffic is high.	Individual	Narrative	Photo

Photo only	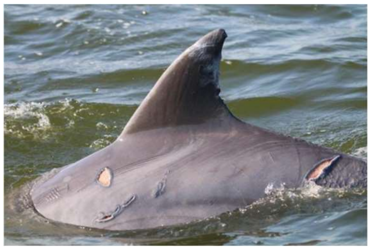	Individual	n/a	Photo

Supplementary figure 1. Correlation plot between variables of interest in Study 1 (a) and Study 2 (b). The color and size of each dot denotes the size and direction of the correlation coefficient r. Correlations that did not reach significance (*p* > 0.05) when correcting for multiple comparisons are crossed out.

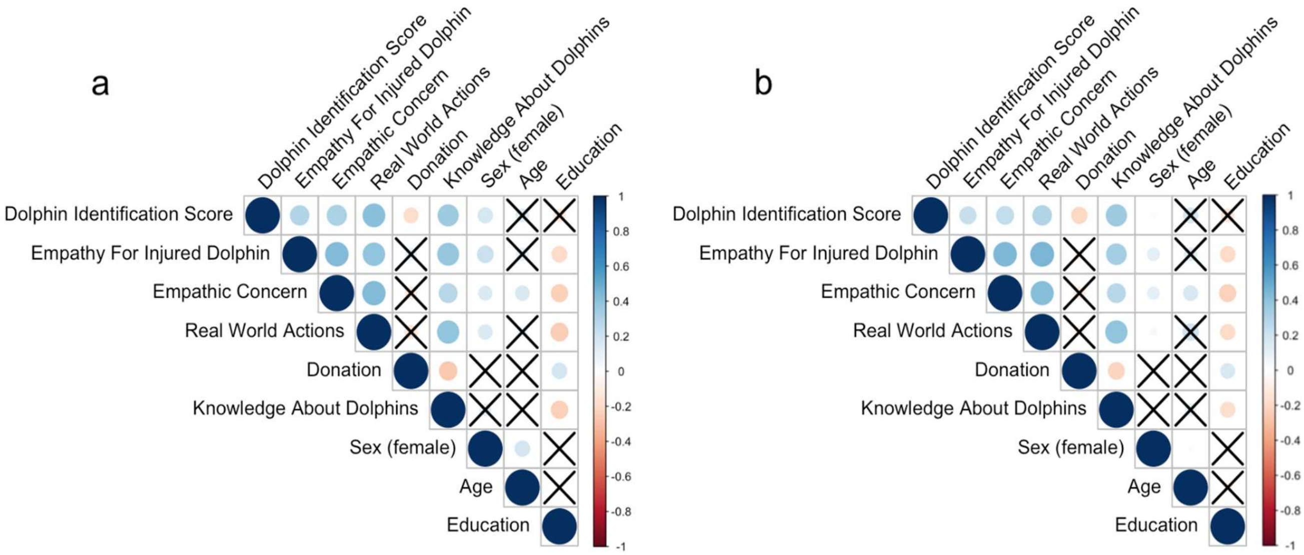

Supplement: Supplementary file 1 — Supplementary file1 (PDF 420 KB) [file 13280_2024_2017_MOESM1_ESM.pdf]
